# What is the impact of biologic therapies on common co-morbidities in patients with rheumatoid arthritis?

**DOI:** 10.1186/s13075-016-1176-x

**Published:** 2016-12-01

**Authors:** Jenny Humphreys, Kimme Hyrich, Deborah Symmons

**Affiliations:** 1 Arthritis Research UK Centre for Epidemiology, University of Manchester, Manchester, UK; 2 NIHR Manchester Musculoskeletal Biomedical Research Unit, Central Manchester NHS Foundation Trust, Manchester Academic Health Science Centre, Manchester, UK; 3 Arthritis Research UK Centre for Epidemiology, Manchester Academic Health Science Centre, University of Manchester, Stopford Building, Oxford Road, Manchester, M13 9PT UK

## Abstract

Biologic therapies have revolutionised disease control in patients with rheumatoid arthritis (RA). Theoretically, they have the potential to influence co-morbid disease associated with RA through better control of systemic inflammation. Conversely, co-morbidity may occur as an adverse effect of the drugs. The latest evidence from observational data shows an increased risk of infection in the first 6 months of treatment with tumour necrosis factor inhibitor (TNFi) therapies and potentially other biologic therapies. Rates of infection after the first 6 months decrease and become comparable to patients with RA treated with conventional synthetic disease modifying anti-rheumatic drugs (csDMARDs). TNFi also appear to reduce the risk of cardiovascular disease in these patients, in particular ischaemic heart disease. TNFi treatment may be associated with a small increase in the risk of developing squamous cell carcinoma of the skin; in terms of other cancers, rates appears to be no different to those seen in patients treated with csDMARDs. There is a paucity of data on the impact of other biologic therapies and the effect of all biologic therapies on other common co-morbidities.

## Background

Biologic therapies, or biologic disease modifying anti-rheumatic drugs (bDMARDs), have transformed disease control and outcomes for patients with rheumatoid arthritis (RA) since they were introduced early this century. RA is a systemic inflammatory disorder of the immune system which predominantly affects the joints. However, it also affects other body systems either directly or indirectly [1]; thus, the impact of bDMARDs is not confined to the joints. Infections [2, 3] and certain types of cancer [4] occur more frequently in patients with RA. Similarly, both fatal and non-fatal cardiovascular diseases (CVD) are approximately 1.5–2-fold more common in patients with RA than in the general population [5, 6]. The increased mortality seen in RA is thought to be driven in a large part by co-morbidity, in particular cardiovascular disease (CVD) [5]. It has been suggested that many of these co-morbidities are a consequence of a high cumulative burden of systemic inflammation [7, 8]. Therefore, it might be hypothesised that better disease control achieved through new, more potent treatments may reduce co-morbidity. Further, some of these co-morbidities (in particular infections and CVD) may be exacerbated by glucocorticoid use, and better disease control may enable reduction in glucocorticoid usage in these patients. On the other hand, co-morbidity in patients with RA may occur as an adverse effect of medication, in particular immune suppression. Thus, bDMARDs might be associated with an increased incidence of some diseases.

The European League Against Rheumatism (EULAR) has recently highlighted six key comorbidities for systematic screening in routine care [9]: infections, CVD, malignancy, gastrointestinal disease, osteoporosis and depression. In this review we explore the evidence for an association between biologic use and a change in any of these co-morbidities. Randomised controlled trials (RCTs) provide relatively limited information because of their strict inclusion and exclusion criteria (often excluding patients with prevalent co-morbidity) and short duration. We therefore focus on observational, real world data. Most published evidence relates to infection, CVD and cancer. As tumour necrosis factor inhibitors (TNFi) were the first bDMARDs to enter clinical practice, they have been the most widely studied biologic drugs.

### Infection

Serious infections (SIs) are infections which lead to hospitalisation, intravenous antibiotics or death. As a disease of dysregulated immune function, RA is associated with an increased risk of SI [10–12]. Glucocorticoids and conventional synthetic DMARDs (csDMARDs) have broad immunosuppressive properties and also predispose to SIs [10, 12–14]. Observational data suggest that the baseline risk of SI in biologic-naïve patients with RA is approximately double that of the general population [11, 12, 15]. BDMARDs target key cytokines and cells involved in both maintaining inflammation and fighting infection. Therefore, it is likely that bDMARDs will increase the risk of SI. Conversely, longer term use of bDMARDs might reduce infection risk by lowering the patient’s requirement for glucocorticoids.

#### Tumour necrosis factor inhibitors

Individual early randomised controlled trials (RCTs) of TNFi did not show a statistically significant increased risk of SI. However, a meta-analysis of nine RCTs of adalimumab (ADA) and infliximab (INF), published in 2006, found a doubling of risk of SI compared to the control arms of the trials [16]. The most recent systematic review and meta-analysis of 106 RCTs of nine biologic therapies found an odds ratio of 1.31 (95% confidence intervals (CI) 1.09, 1.58) for standard dose bDMARDs versus conventional synthetic disease modifying anti-rheumatic drug (csDMARDs) [17], which translated to an additional six SIs per 1000 person-years in patients taking bDMARDs compared to csDMARDs alone. There was no apparent difference in risk between bDMARDs.

The latest systematic review of observational data identified nine studies of SI in patients treated with TNFi. Adjusted hazard ratios (HRs; compared to csDMARDs) ranged from 1.1 to 1.8 [18]. Some of the variation between studies can be attributed to varying periods of follow-up or ‘time at risk’ [19]. It is now established that the highest risk is within the first 6 months of therapy with a gradual decline thereafter [19–21]. Around two-thirds of the decline can be attributed to ‘depletion of susceptibles’, i.e. patients who experience an SI then stop TNFi therapy, and one-third to an improvement in physical function and a decrease in steroid dosage [22]. The most common sites of SI are the lower respiratory tract followed by skin and soft-tissue [20, 23].

Not all RA patients treated with TNFi are at the same risk for SI. The German Biologics Register (RABBIT) found that treatment with ≥7.5 mg glucocorticoid daily, age greater than 60 years, chronic renal or lung disease, more than five previous csDMARD failures and previous SI were risk factors for the development of SI [22]. Interestingly, the same register has very recently shown that the risk of both sepsis and mortality following an SI is lower in patients with RA exposed to bDMARDs compared to csDMARDs [24].

##### Tuberculosis

A case of tuberculosis (TB) was reported in a phase III trial of INF in 1999 [25]. An increased risk of TB and other granulomatous diseases was identified in patients treated with the monoclonal antibodies INF and ADA, initially through spontaneous reporting to national pharmacovigilance programmes [26], then subsequently in the Spanish BIOBADASAR [27] and other observational registers (summarised in [28]). Most cases of TB occurred within 6 months of starting INF, suggesting re-activation of latent TB. The British Society for Rheumatology Biologics Register for Rheumatoid Arthritis (BSRBR-RA) reported 40 cases of TB following TNFi (crude incidence rate 118/100,000 person-years); 62% were extra-pulmonary and ten resulted in death [29]. Although rates were higher in INF- and ADA-treated patients, cases did occur in etanercept (ETN)-treated patients. This and other reports led to the introduction of a number of national guidelines for screening for and treatment of latent TB with a subsequent reduction in the rates of TB [30]. Data from RCTs suggest that the risk of TB may be higher in certolizumab pegol- and golimumab-treated patients [28]; however, most cases came from countries with a high background prevalence of TB. Results from observational data are awaited but clearly the background risk of TB is an important consideration when making decisions about biologic therapy in RA.

There is also a small increase in risk of other opportunistic infections, including legionellosis, non-tuberculous mycobacteria, listeriosis, salmonellosis and nocardiosis. As with SI and TB, the risk is highest in the early months after TNFi initiation [31].

##### Herpes zoster

The risk of reactivation of herpes zoster (HZ; shingles) is increased in patients with decreased cell-mediated immunity. An increased risk of HZ has been reported by the German Biologics Register [32], the US Veteran’s Administrative Database [33] and the BSRBR-RA [34]; further, a 1.6-fold increase in risk of HZ was found in a systematic review and meta-analysis summarising published data up to March 2013 [35]. However, a study of 36,212 RA patients (24,384 initiating TNFis) from four US automated databases found no difference in the risk of HZ in patients initiating TNFi compared to those initiating csDMARDs after adjusting for propensity score [36]. Increasing age and glucocorticoid dose greater than 10 mg daily were additional risk factors for HZ in all these studies [28, 36].

#### Other bDMARDs

Less is known, particularly from observational studies, about the infection risk associated with non-TNFi bDMARDs. The most recent systematic review and meta-analysis of RCT data suggests that the overall risk of SI does not differ between biologic therapies when used in standard doses, but is approximately 30% higher than in patients treated with csDMARDs alone [17]. A recent observational study of US veterans with RA found similar rates of SI in patients treated with rituximab (RTX) or abatacept (ABT) to ETN [37]. RTX acts by depleting pre-B cells and as a consequence leads to reduced levels of IgG. Approximately 4% of patients develop persistent hypogammaglobulinaemia following repeated courses of RTX [38]. These patients, who tend to be older with more co-morbidity, have a higher risk of SI.

There is no signal of an increased risk of TB with RTX, ABT or tocilizumab (TCZ) within the context of RCTs. There may be a slight increase in the risk of progressive multifocal leukoencephalopathy in patients treated with RTX [39].

### Cardiovascular disease

CVD is increased in patients with RA [5–7, 40]. This association is partly attributable to an increased prevalence of traditional cardiovascular (CV) risk factors such as smoking [41], abnormal lipid profiles [42] and high body fat:muscle mass ratio [43]; partly to shared genetic risk factors [44, 45]; and partly to the effect of systemic inflammation on the vasculature [40, 46, 47]. This link between inflammation and atherosclerosis suggests CVD risk may be particularly responsive to RA disease control. There are data to suggest that successful disease control improves surrogate markers of CVD, such as endothelial dysfunction and carotid intimal media thickness [48–50]. A recent study using data from the Consortium of Rheumatology Researchers of North America (CORRONA) found that a ten-point reduction in the Clinical Disease Activity Index (CDAI) [51] over a median of 2.7 years was associated with a 21% reduction in risk of CV events [52]. Similarly, a recent nested case–control study from Germany demonstrated that levels of systemic inflammation, measured by C-reactive protein and erythrocyte sedimentation rate, were higher in myocardial infarction (MI) cases compared to controls and also that treatment of CV comorbidity was lower in MI cases [53]. Another, much smaller, case–control study from Germany of patients with RA and heart failure demonstrated that patients with heart failure had more active RA than controls [54]. There may be treatment specific effects; for example the use of methotrexate (MTX), the anchor csDMARD in the treatment of RA, has been associated with up to 70% reduction in mortality and, in particular, CV mortality [55]. In addition some data suggest effective treatment with any csDMARD reduces the risk of CVD [56].

#### Tumour necrosis factor inhibitors

A systematic review and meta-analysis, published in 2011, of observational cohort data found an overall 54% reduction in the risk of all CV events in TNFi users compared to patients taking csDMARDs [57]. National drug registers in both Sweden and the UK have reported a reduction in risk of CVD in patients who show a good response to TNFi therapy compared to non-responders [58, 59]; in addition, longer duration of therapy appears to reduce the risk of CV events [60]. A recent study from the BSRBR-RA in the UK, which linked registry data to a national audit of MI for event verification, demonstrated that patients with RA taking their first TNFi had a 40% decrease in rates of MI compared to propensity score-matched csDMARD users [61]. Interestingly, in similar analyses, rates of stroke were equivalent in TNFi users compared to those taking csDMARDs (HR 0.99; 95% CI 0.54, 1.81) [62]. This may be because strokes are less common than MIs and so the study lacked the power to detect a difference; alternatively, it may be that improved RA disease control does not translate to fewer strokes or that any improvement is balanced out by an increased risk conferred by the drugs themselves.

Reports on the association between TNFi therapy and heart failure have been conflicting. After finding increased levels of TNF in patients with heart failure, trials were conducted using TNFi as treatment for heart failure [63, 64]; none demonstrated benefit and one reported worse outcomes in patients treated with TNFi [64]. Consequently, a ‘black box’ warning was introduced not to use these medications in patients with pre-existing heart failure. An early study in patients with RA suggested a reduction in the rates of new onset heart failure in TNFi users [65], but later data showed a non-statistically significant trend towards increased rates of incident heart failure [66]. The most recent data from the US suggest similar rates of heart failure in new TNFi users and new users of csDMARDs (HR 0.85; 95% CI 0.63 to 1.14) but identified that such a difference may have existed prior to the introduction of the black box warning in 2002 [67]. This study also noted a dose-dependent association between glucocorticoid use and heart failure. Importantly, however, the authors acknowledged that they were unable to adjust for potential differences in baseline disease severity between the TNFi and csDMARD groups as this information was not collected. The German RABBIT register also found similar rates of heart failure in TNFi compared to csDMARD users [68] and a dose-dependent association with glucocorticoids. The authors of that study suggest that the overall effect of TNFi is more beneficial (through improved control of disease activity and reduced need for glucocorticoids) than harmful.

#### Other bDMARDs

Observational data are limited regarding the relationship between non-TNFi bDMARDs and CVD in patients with RA. The largest study to date used administrative claims data in the US to compare rates of CVD between patients with RA taking different bDMARDs [69]. They included over 47,000 patients and found rates of CVD were higher in patients treated with TNFi compared to ABT (HR 1.28; 95% CI 1.04 to 1.56). A number of other studies have provided some potential data but are insufficient to draw firm conclusions. A study from the German RABBIT registry found improved all-cause mortality in RTX users compared to csDMARD users [70]. This study did not examine CV mortality specifically; however, given that it is the most common cause of mortality in RA, we might infer a possible effect of RTX on CVD. Some small pilot studies have shown beneficial effects of RTX on endothelial dysfunction and lipid profiles [71, 72]; conversely, the reduced circulating IgM levels associated with long-term treatment with RTX may confer a pro-atherogenic effect [73]. Particular concerns exist regarding CV event rates in patients receiving TCZ, as IL-6 blockade is associated with increased levels of total cholesterol and high-density and low-density lipoproteins [74]. However TCZ, has only been widely available in clinical practice since 2010 and therefore insufficient observational data have accumulated to address this question. A post hoc analysis of pooled data from RCTs and open label extension studies found that higher levels of disease activity and joint counts amongst patients treated with TCZ were associated with CV events but changes in lipid parameters were not [74]. A very small study from Norway identified improvements in pulse wave velocity (another surrogate marker for CVD) in 36 patients taking RTX and TCZ over 12 months, with no change in patients taking ABT [75]. However, given the small number of patients and limited follow-up, it is difficult to draw strong conclusions from these data about long-term CV effects.

### Cancer

Patients with RA have an increased risk of cancer compared to the general population, with a meta-analysis published in 2015 by Simon et al. [76] showing a small, statistically significant increase of 9% in the overall risk of malignancy in patients with RA.

In terms of specific cancers, an increased risk of lymphoma is well recognised in patients with RA [77]. This risk is greatest in patients with the highest cumulative burden of disease activity [8]. The type of lymphomas seen in patients with RA suggests an underlying immune deficiency profile prone to lymphomagenesis [77]. Even prior to the introduction of TNFi therapies, some lymphoma events were linked to the use of csDMARDs, in particular cyclophosphamide, azathioprine and MTX, in the treatment of RA [78]. The meta-analysis by Simon et al. found increased rates of lymphoma as expected, but additionally identified a greater risk of lung cancer (which may in part be accounted for by smoking, a shared risk factor for RA and lung cancer [79]) and a decreased risk of colorectal cancers in patients with RA compared to the general population. A study from the UK, published in 2013, also identified an increased incidence of lymphoma and lung cancer but showed a reduction in prostate and cervical cancers in patients with RA [80]. In an administrative claims database in South Korea, there was again an increased risk of lymphoma but lower rates of gastric cancer [81].

A number of studies have found an increased occurrence of non-melanoma skin cancer (NMSC) in RA compared to the general population [82–84]. By contrast, it is unclear whether RA is associated with malignant melanoma. There is a well-documented link between the immunocompromised host and melanoma, with evidence from patients with AIDS and post organ transplant [85, 86]. However, data in patients with RA taking csDMARDs have been inconsistent [80, 87]. Interestingly, a study from Australia, which has the highest background incidence of melanoma worldwide, found an increased risk of melanoma in MTX users compared to the background population [88]. In the meta-analysis by Simon et al. [76] the pooled standardised incidence rate for melanoma was significantly higher in RA patients, although the authors noted that few of the individual studies found a statistically significant increased risk.

#### Tumour necrosis factor inhibitors

As TNF is known to have anti-tumour effects, there were initial concerns that TNF inhibition could increase cancer incidence [89]. Data on incident malignancies in the TNFi RCTs were mixed, with some earlier individual trials and meta-analyses appearing to show an increased risk of cancer [16, 90]. However, more recent systematic reviews and meta-analyses have not been able to replicate this finding [84, 91]. A systematic review by Askling et al. [91], published in 2011, explored the potential cause of these different results. They noted that previous meta-analyses did not include all of the published trials, that the number of events in both treatment and control arms was low and that the definitions of cancer across individual studies may not be comparable. They attempted to address these issues in their meta-analysis. The authors were provided with patient level data from each study sponsor with more detailed information on study design, patent information and treatment details than was available in the published reports. Cancer events were identified by a search of each study sponsor’s clinical and safety database, which allowed the same definition of a cancer to be used across all studies. They found no difference between cancer rates across the three TNFi studied (INF, ETN and ADA), but did find differing rates of cancer in respective control arms.

As a result of the initial concerns, patients with a history of prior cancer were excluded from the majority of TNFi RCTs and, post-licensing, TNFi therapy was felt to be contra-indicated in patients with a previous malignancy. Therefore, information is limited on the recurrence of cancer in patients with prior malignancy treated with TNFi. Nevertheless, recent studies from administrative claims databases in the US and the Swedish ARTIS register have reported no increase in recurrent cancers in TNFi-treated versus biologic-naïve patients with similar history of prior malignancy [92, 93]. These studies are also supported by data from the British and German registries [94, 95]. Notably, all studies were based on small numbers of cases.

Regarding specific malignancies, the risk of lymphoma in TNFi users has been widely studied. However, as lymphoma is relatively rare, studies have struggled to identify sufficient cases of lymphoma to reach robust conclusions. Observational studies from the UK, the US and Sweden have examined the potential associated between TNFi and lymphoma in patients with RA [4, 96–99]. A study from South Sweden, published in 2005, reported a large, but non-significant, relative risk of lymphoma of 4.9 (95% CI 0.9, 26.2) in patients treated with TNFi compared to csDMARDs. Two US studies using the National Data Bank for Rheumatic Diseases (NDB), found similar rates of lymphoma in TNFi users compared to other patients with RA [4, 97]. However, the Swedish and US studies were limited by potential confounding by indication, i.e. patients with more severe disease are more likely to be prescribed TNFi. This was not adjusted for at all in the first NDB publication [4]. The Southern Swedish and second NDB publication adjusted for the baseline Health Assessment Questionnaire score [97], which is more a measure of disability than disease activity and therefore there may have been residual confounding. The BSRBR-RA study used propensity score matching to account for confounding by indication and other imbalances between baseline covariates between TNFi- and csDMARD-treated patients; they reported an equivocal HR for lymphoma of 1.00 (95% CI 0.56, 1.80) [96]. The national Swedish ARTIS registry also found no increased risk of lymphoma in TNFi-exposed compared to csDMARD-exposed patients [98]. Nevertheless, all of these studies remain potentially underpowered, either through insufficient person-years of observation or the low number of events. A recent EULAR initiative performed a joint analysis with data from 11 registers which has been published only as an abstract so far [100]. With some 600,000 patient-years of exposure no increased risk was found. The results are reassuring not only concerning the overall incidence of lymphomas but also the spectrum of subtypes.

Data from national European registries in the UK, Germany and Denmark have consistently demonstrated no increased occurrence of solid cancers in patients treated with TNFi versus those treated with csDMARDs [95, 101, 102]. These data are also supported by an older study from the NDB in the US [103].

The risk of skin cancer appears to be unaltered by TNFi therapy. A meta-analysis of observational and long-term extension studies in 2012 by Le Blay et al. [84] found no increase in NMSC in TNFi users compared to csDMARD users (pooled odds ratio 0.79; 95% CI 0.62, 1.02). Subsequent studies from the DANBIO registry in Denmark and the BSRBR-RA also found no difference in incidence of NMSC rates between TNFi and csDMARD users [83, 102]. However, the most recent study from Sweden, the largest to date with over 2800 NMSCs, did identify a small increase in the risk of squamous cell carcinoma (SCC), but not basal cell carcinoma, in TNFi users compared to those taking csDMARDs [82]. The studies from Denmark and the UK comprised mostly basal cell carcinomas and relatively few SCCs and so may have been underpowered to detect a small increase in SCC. The EULAR initiative mentioned above also pooled data from 11 observational registries across Europe in order to explore the risk of malignant melanoma in TNFi versus csDMARD users [104]. This study captured data on over 130,000 patients over close to 580,000 patient-years follow-up. The overall standardised incidence ratio, adjusted for calendar year, and age and sex standardised to the reference population of each country, was higher in the ever TNFi-exposed versus biologic-naïve patients at 1.1 (95% CI 0.9, 1.4), but this did not reach statistical significance.

#### Other bDMARDs

A small number of studies have investigated the risk of malignancy in patients treated with other bDMARDs. Simon et al. [105] compared rates of malignancy in the RCT data for ABT with rates seen in five observational cohorts of patients with RA taking csDMARDs. They found the standardised incidence ratios were no different in the ABT user group. Similarly, Solomon et al. [106] investigated the risk of malignancy in a range of biologic and csDMARD users compared to MTX users and found lower rates of malignancy in TNFi users but equivalent rates (with wide confidence intervals) in patients treated with RTX and ABT. Scott et al. [107] examined rates of recurrent NMSC in patients with RA or inflammatory bowel disease across a number of immunosuppressive therapies. Rates of recurrence were similar in ABT and RTX users compared to MTX users. The number of events in each treatment group was small and may have been underpowered to detect a true difference. Similarly, the collaborative European project described above found no increase in the risk of malignant melanoma in RTX, ABT and TCZ users compared to biologic-naïve patients [104]; again, both the number of events and total follow-up time were small.

### Other comorbidities: gastrointestinal disease, depression, osteoporosis

Gastrointestinal (GI) disease is an important co-morbidity in RA partly due to side effects of older medications. Non-steroidal anti-inflammatory drugs (NSAIDs) and glucocorticoid use are associated with peptic ulcer disease and GI perforation [108, 109]. A number of registries have investigated the effect of TNFi and other bDMARDs on GI perforation [109–111] with no association identified, although this outcome is rare and the individual studies are probably underpowered. Curtis et al. [109] noted that 70% of the patients who experienced a GI perforation were either taking glucocorticoids or had a history of diverticular disease; similarly, in the BSRBR-RA the relative risk for GI perforation was nearly three times greater in patients taking glucocorticoids compared to those who were not [110]. Van Vollenhoven et al. [112] summarised the risk of diverticular perforation in RCTs of TCZ. The reported rates appeared to be slightly higher than rates seen from other studies with TNFi and csDMARDS [113]. Although this study was published in abstract form only, recent observational data from two studies also support an increased risk of GI perforation in patients treated with TCZ compared to those treated with other bDMARDs and csDMARDs [107, 114]. 

Data are very limited on the relationship between bDMARDs and depression. Depression is common in patients with RA [115–117] and is higher compared to the general population [118]. In these patients, depression is associated with levels of pain, disability and fatigue [116, 119, 120]. Few studies have investigated the impact of treatment of RA on depression. A study from the BSRBR-RA examined the longitudinal relationship between TNFi use and fatigue [119]. They found that, although fatigue improves following treatment [119], a large proportion of patients report residual fatigue despite clinical remission [121]. A meta-analysis of RCT data found no difference in levels of fatigue between patients treated with TNFi and those treated with csDMARDs [122]. However, although depression and fatigue are similar constructs, they are not identical and thus we cannot make direct inferences about depression from these findings. A biological link has been postulated between increased levels of IL-6 and depression [123]. However, trials of TCZ as a treatment for depression have yet to be conducted and there are no observational data to support such a link.

Again, data are limited on the relationship between bDMARDs and bone health. RA is a recognised risk factor for the development of osteoporosis and both longer disease duration and treatment with glucocorticoids enhance that risk [124–126]. A few small studies have suggested bone remodelling may improve with TNFi use [127–129]. However, larger observational studies have failed to find a reduction in fracture rates or improvement in bone density in TNFi users versus non-users. Coulson et al. [130], using data from the CORRONA registry, found that incident fracture rates were not associated with TNFi or csDMARD treatment, although they were higher in glucocorticoid users compared to non-users. Roussy et al. [131] investigated the impact of bDMARDs (including TNFi, RTX, ABT and anakinra) in a nested case control study of a Canadian claims database. As with the CORRONA study, they found no evidence of an association between sustained recent exposure to biologic drugs and non-vertebral fracture rates.

## Conclusions

The impact of biologic therapies on outcomes for patients has been substantial in terms of their disease control. In addition it appears that TNFi at least can improve some of the burden of comorbidity associated with the disease, particularly CVD. In terms of de novo cancer, results have also generally been reassuring. There may be a small risk of SCC of the skin in patients taking bDMARDs for the first time [82], but this remains so far an isolated signal. Studies of lymphoma remain underpowered. However, if a strong signal existed it should have been identified by the existing literature and therefore it is reassuring that any increased risk from TNFi, if present, is likely to be small. Equally, the small number of studies which have investigated recurrence of cancer suggest TNFi may be safe in this population. There is an increased risk of serious infections in the first 6 months of treatment with TNFi (and probably all bDMARDs), but this diminishes after this time for those who remain on treatment.

There remains a paucity of data on the impact of TNFi on other common comorbidities such as depression, GI disease and osteoporosis. Further, newer biologic agents with different mechanisms of action to TNFi, such as RTX, ABT and TCZ, have not been studied in sufficient detail to draw firm conclusions about their effects.

## Abbreviations

ABT: Abatacept
ADA: Adalimumab
bDMARD: Biologic disease-modifying anti-rheumatic drug
BSRBR-RA: British Society for Rheumatology BDMARDs Register for Rheumatoid Arthritis
CI: Confidence interval
CORRONA: Consortium of Rheumatology Researchers of North America
csDMARD: Conventional synthetic disease modifying anti-rheumatic drug
CV: Cardiovascular
CVD: Cardiovascular disease
ETN: Etanercept
EULAR: European League Against Rheumatism
GI: Gastrointestinal
HR: Hazard ratio
HZ: Herpes zoster
INF: Infliximab
MI: Myocardial infarction
MTX: Methotrexate
NDB: National Databank for Rheumatic Diseases
NMSC: Non-melanoma skin cancer
RA: Rheumatoid arthritis
RCT: Randomised controlled trial
RTX: Rituximab
SCC: Squamous cell carcinoma
SI: Serious infection
TB: Tuberculosis
TCZ: Tocilizumab
TNFi: Tumour necrosis factor inhibitor
